# Erratum: Training Non-Oncologist Healthcare Providers to Manage Oral Endocrine Therapy for Breast Cancer

**DOI:** 10.1200/GO-25-00420

**Published:** 2025-08-19

**Authors:** Aimable Ndayishimiye, Alexandra Fehr, Vestine Rugema, Vincent Kalumire Cubaka, Jean Claude Niyibizi, Francois Sebishyimno, Aline Umwizerwa, Nicaise Nsabimana, Cyprien Shyirambere, Temidayo Fadeli

The meeting abstract by Ndayishimiye et al titled “Training Non-Oncologist Healthcare Providers to Manage Oral Endocrine Therapy for Breast Cancer” (*JCO Glob Oncol* 10.1200/GO-24-14300) was published online July 26, 2024, with errors.

In the author list and any subsequent occurrences, “Temidayo Fadeli, MPH” should have been “Temidayo **Fadelu, MD**, MPH.”

Temidayo Fadelu's listed affiliation was “Partners in Health, Rwinkwavu, Rwanda” but should have been “**Dana-Farber Cancer Institute, Boston, MA**.”

This has been corrected as of August 19, 2025. The authors apologize for the errors.

